# Eating Disorder Focused Family Therapy with Autistic Children and Young People: A Narrative Review of Recent Developments

**DOI:** 10.1007/s11920-026-01710-3

**Published:** 2026-08-14

**Authors:** Amelia Austin, Rachel Loomes, Fiona Duffy

**Affiliations:** 1 Department of Clinical Psychology, School of Health in Social Science, University of Edinburgh, Edinburgh, EH8 9AG UK; 2 Department of Psychology, School of Health and Life Sciences, Glasgow Caledonian University, Glasgow, UK; 3 Department of Clinical, Educational, & Health Psychology, University College London, London, UK; 4 Maudsley Centre for Child and Adolescent Eating Disorders, South London and Maudsley NHS Foundation Trust, London, England, UK; 5 NHS Lothian Child and Adolescent Mental Health Services, Royal Edinburgh Hospital, Edinburgh, Scotland, UK

**Keywords:** Adolescent, Anorexia nervosa, Autism, Eating disorder, Family-based treatment, Family therapy

## Abstract

**Purpose of review:**

This review aims to report the recent advances for eating disorder focused family therapy (FT-ED) for Autistic children and young people, including outcomes, experiences, and suggested adaptations.

**Recent findings:**

Quantitative studies suggest that Autistic children and young people (and those with high autistic traits) receiving outpatient FT-ED are more likely to require escalation to more intensive levels of care compared to non-autistic peers. Qualitative research shows that Autistic young people and their parents/carers often report poor experiences of FT-ED. Clinicians report a lack of confidence, particularly when adapting care from a manualised approach. Commonly suggested adaptations include environmental adjustments (e.g., quiet spaces, dimmed lights), sensory-informed understanding of food and eating preferences (e.g., accounting for historical eating behaviour), communication adaptations (e.g., passports and clear, literal language), psychoeducation on autism and eating disorders, careful consideration of externalization, and use of separated sessions.

**Summary:**

Given that Autistic children and young people and their parents report poorer experiences of FT-ED relative to their non-autistic peers, adaptations that accommodate autistic needs while not interfering with ED recovery should be considered. Further development of guidelines and decision-making tools may support FT-ED clinicians to deliver effective and inclusive care.

## Introduction

Eating disorders (EDs), including anorexia nervosa (AN), are serious illnesses associated with significant physical decline, psychological distress, social deterioration, and mortality [1]. Findings from a recent meta-analysis suggest that approximately 29% of people with AN are Autistic or have high autistic traits [2]. In comparison, about 1–2% of the general population are autistic [3, 4]. Previous research with Autistic adults suggests that many (particularly those with AN) experienced traditional ED treatment to be inadequately tailored to their needs, such as communication and sensory differences [5–7]. This need spurred Tchanturia and colleagues in London in to develop PEACE (Pathway for Eating disorder and Autism Developed from Clinical Experience), starting in 2017, to improve inpatient services for Autistic adults [8–10]. Evaluation of PEACE has demonstrated that it improves service experience [11], clinical outcomes, and shows initial cost effectiveness [12].

For children and adolescents, the recommended treatment approach for AN across international clinical guidelines is ED focused family therapy (FT-ED) [13–15]. FT-ED is an overarching term used to describe approaches to treatment which derive from Dare and colleagues’ family therapy for AN (FT-AN) at the Maudsley Hospital in London in the 1980s [16]. It includes manualised family-based treatment (FBT) [17] and FT-AN, which has continued to be developed by the Maudsley team [18, 19]. Within this paper, we will refer to specific treatment approaches when used in the original papers or FT-ED when referring to these approaches collectively. While there are some differences between FT-ED approaches for AN (e.g., dietetics involvement, use of formulation), there are many commonalities [20, 21]. Key techniques used within FT-ED include agnosticism, or the focus of moving forward rather than on determining the cause of the illness [22], and externalisation, or referring to the ED as if it is separate from the young person, often using imagery and metaphor [22]. FT-ED also make use of psychoeducation, which is the provision of knowledge around an illness [23].

FT-ED takes a phased approach, with an initial focus on weight restoration and normalising eating patterns. Parents and carers are seen as a key resource and are tasked with the renourishment of their child. As the young person begins to regain physical health, the goal of treatment shifts to handing back age-appropriate responsibility for eating. The later stage of FT-ED explores the family lifecycle and adolescent development issues [17, 19]. Meta-analytic evidence suggests that FT-ED is significantly superior to an individual therapy approach for adolescents with AN when immediate weight gain is paramount [24]. However, it is still considered to have less than a 50% success rate of achieving full remission [25].

FT-ED, like most traditional ED treatments, did not explicitly consider neurodiversity when being developed and evaluated. Given the mixed acceptability and effectiveness of current psychotherapies with Autistic adults experiencing EDs (see Nimbley et al. [26]), it is important to consider how FT-ED is working—or not working—for Autistic young people and their families. The aim of the current review is to narratively synthesise the current evidence, both peer-reviewed articles and grey literature (e.g., theses/dissertations), on FT-ED with Autistic children and young people. A pragmatic search of published and unpublished databases/sources (PubMed, Open Access Theses and Dissertations, ResearchGate, Google Scholar, PsyArXiv) was conducted up to May 2026, with search terms related to autism, EDs, and FT-ED. Evidence on FT-ED with Autistic children and young people is presented below in three sections: (1) treatment use and outcomes, (2) treatment experiences of parents/carers, young people, and clinicians, and (3) suggested treatment adaptations. We then outline our perspectives on limitations and future directions.

## Treatment Use and Outcomes

Four quantitative studies have reported on the relationship between FT-ED and treatment use/outcomes in Autistic children and young people [27–30]. Stewart and colleagues [27] performed a clinical audit at the Maudsley Centre for Child and Adolescent Eating Disorders, reviewing the outcomes for girls aged 9–18 who received outpatient FT-AN between 2009 and 2015. High levels of autistic traits were determined based on the Autism Quotient (AQ), using a cut-off score of 30 [31]. Of the 289 eligible patients, 20 (6.9%) scored above the cut-off score, indicating higher autistic traits. There was no difference in the rates of physical recovery (based on the Morgan and Russell [32] criteria) between those with higher autistic traits and those with lower autistic traits. However, the group of patients with high autistic traits were significantly more likely to need more intensive services, such as day programme or inpatient care, in comparison to patients in the group with lower autistic traits (χ^2^ = 7.30, df = 1, *p*<.01).

Bentz and team [28] in Denmark examined outcomes for Autistic and non-autistic young people age 11 to 17 referred for FBT for AN in outpatient services. Across a span of 16 months, 157 young people were referred, 16 (10.2%) of whom were diagnosed Autistic. Comparing outcomes for Autistic and non-autistic peers, there were no significant differences in rates of weight restoration, treatment completion, or time to remission. However, significantly more young people in the Autistic group (50%) needed more intensive care in comparison the non-autistic group (16%). Bentz and colleagues [29] went on to examine predictors of response to outpatient FBT for 653 Danish children and adolescents with AN between the ages of 7 and 17. Of the 21% (*n* = 136) who were diagnosed Autistic, the median treatment time was 17.6 months (compared to 11.6 months for non-autistic peers). This equated to a 66% risk that Autistic children and adolescents would still be in FBT beyond 12 months. Autistic young people also had a 32% chance of needing more intensified treatment in comparison to a 15% chance for non-autistic peers.

In Italy, Pruccoli and colleagues [30] examined the use of an FT-AN based inpatient program in 82 adolescents aged 12–17 years. Twenty-two patients were deemed to have higher autistic traits based on scores from the AQ and the Autism Diagnostic Observation Schedule-Second Edition (ADOS-2) [33]. Results showed that treatment intensity (number and duration of admissions, use of psychotropic medication) and discharge percentage body mass index (BMI) did not differ between those with higher autistic traits compared to peers with lower autistic traits.

## Treatment Experiences

Five qualitative studies have reported on the experiences of Autistic young people, their parents, or clinicians with FT-ED [34–38]. Four additional descriptive or qualitative papers [39–42] were broader in scope (e.g., examined ED services for Autistic young people) but provided data that was relevant to lived experiences of FT-ED.

### Parent Experiences

Research generally reported poor experiences of FT-ED for parents of Autistic children and young people. Several studies have documented the emotional toll of FT-ED on parents of Autistic young people, including feelings of frustration, isolation, abandonment, and personal mental health decline [34–36, 39]. Loizou [39] found that these feelings may be more prominent during the initial stages of treatment. Alford’s [40] exploration of parents of Autistic children’s experiences in ED services, including FT-ED, suggests an overarching experience of being misunderstood due to factors such as siloed services and expertise, a pathologisation of autistic traits, and a lack of consideration for parental neurodivergence. Nimbley [34] reported parental concerns about rigid adherence to manualised approaches, with some participants feeling as though the protocol was more valued than the patient. However, positive and helpful experiences of FT-ED have also been reported by some parents of Autistic young people, particularly when adaptations have been considered [35]. Across the qualitative literature, there was a strong parental desire for adaptations, with parents often reporting that they had to assume an advocacy role for their young person [34–36, 39].

### Young Peoples’ Experiences

There is also emerging research describing the experiences of Autistic young people in FT-ED. Haugaard [37] reported that some Autistic young people partaking in FT-ED felt sidelined during the initial phase of treatment, with its strong focus on mobilising parents to renourish their child. Some young people also reported that FT-ED left little room for them to openly express themselves and avoided sharing in family sessions to prevent scrutiny. Further, some young people reported that recovery expectations were centered around neurotypical norms, such as expectations that interoceptive awareness of hunger cues would return once they were weight restored [37]. Work by Alford [40] found that some Autistic young people who received ED treatment reported it to feel like a one size fits all approach that was misattuned to autistic needs; for example, one young person felt FT-ED attempted to override autistic traits like a drive for autonomy [40]. Across studies, when adaptations were implemented, this was generally reported to improve young peoples’ experiences [37, 40, 41]. Finally, other young family members may also be affected: parents in one study reported that they were so stretched in supporting their Autistic young person through AN that it felt impossible to continue parenting their other children [36].

### Clinician Experiences

Duffy and team’s [38] work exploring the experiences of clinicians delivering FT-ED to Autistic young people and their families reported a theme of reduced clinician confidence, particularly in differentiating between the autism and an ED. Many FT-ED clinicians felt a tension when attempting to adapt FT-ED, feeling pressure to stick to a manualised approach, but also experiencing worry about causing harm by adapting—or not adapting—treatment. Further work by Nimbley [42] found that a key barrier to autism-affirming care in FT-ED was clinician anxiety that adaptations to manualised care would not be considered evidence-based practice.

## Treatment Adaptations

Five qualitative studies [34, 35, 37, 38, 43] and one expert clinical opinion paper [44] explored potential adaptations to FT-ED for Autistic young people. Four additional descriptive or qualitative papers [39–41, 45] were broader in scope (e.g., examined ED services for Autistic young people) but provided data that was relevant to the adaptation of FT-ED.

### General Approach/principles

Multiple studies called for a more autism-affirming and strengths-based approach within FT-ED [34, 37, 38, 40, 43, 44]. Examples included having the young person share their autistic experience [38], harnessing autistic strengths, such as preference for routine and predictability into FT-ED [34], and incorporation of any interest that the young person is very passionate about into assessment or treatment [37, 40, 44]. This strengths-based approach also extends to parents and carers, who offer extensive expertise on which approaches historically work, or don’t work, for their Autistic child [38, 43].

### Sensory and Communication Adaptations

Many of the adaptations described in the literature focus on improving the accessibility of FT-ED for Autistic children and young people by addressing sensory and communication differences. Qualitative research and expert clinical opinion broadly support sensory adaptations to the physical environment in FT-ED [34, 35, 37, 43, 44] and for child and adolescent ED services delivering FT-ED in addition to other modalities and levels of care [40, 41, 45]. This includes the assessment and consideration of the sensory environment, and provision of accommodations such as dedicated quiet spaces or fidgets, and adjusting lighting and smells [37, 40, 41, 43–45]. This also extends to the assessment and adaptation for food related sensory needs, such as adapting meal plans by considering foods that were avoided prior to ED onset, focusing less on food variety in the early stages of FT-ED, and allowing the use of preferred eating utensils [34, 35, 40, 43, 44].

Beyond sensory considerations, the literature highlights the importance of adapting communication within FT-ED to align with autistic information-processing styles, with support from qualitative research and expert clinical opinion in both FT-ED specifically [35, 38, 43, 44] and broader child and adolescent ED services delivering FT-ED alongside other care [40, 41, 45]. One approach to this is the use of a communication passport—a one page document outlining preferences, challenges, dislikes, and support needs [46]—and the flexible use of communication tools [40, 41, 43, 45]. During FT-ED sessions, pragmatic and direct communication, in contrast to guided exploration may work best for some Autistic family members [38, 40, 43], and the use of figurative language, idioms, or circular questioning may not be aligned with autistic cognitive profiles [38, 43, 44].

Some adaptations to communication were made specifically to increase predictability. Several studies highlighted the importance of giving advance notice of any upcoming changes [38, 39, 41, 43, 44], with advance notice of the end of therapy being stressed [39, 44]. One additional suggestion was to describe or provide in written format the agenda for therapy sessions [37, 40]. Loomes and Bryant-Waugh [44] proposed that an initial website with media showing the physical building, describing the assessment process, and introducing team members could be provided prior to initial FT-ED assessment.

Taking into account increased prevalence of neurodivergence in families, existing evidence also supports the consideration of Autistic family members’ needs [34, 35, 38, 40]. This may help to inform communication needs with parents [38], as well as the understanding that Autistic parents or other family members may need to realise the ‘why’ behind treatment approaches before fully getting on board [40]. These considerations are important given the genetic heritability of autism [47], as well as the prevalence of autistic traits in biological parents of Autistic children, which has been estimated between 2.6 and 80% [48].

### Structure and Content Adaptations

A further set of adaptations involves adjustments to the structure, pacing, and content of FT-ED to better support Autistic young people and their families. Several qualitative studies have also suggested that having all family members in the same room at once may not always be ideal. Multiple studies spanning clinicians, parents, and young people described the advantages of a separated FT-ED model [34, 37, 38, 43]. For example, some clinicians in Duffy et al. found that a separated approach could be helpful when aiming to have open conversations around parenting experiences or if the young person is getting overwhelmed within family sessions [38]. Other studies described a similar concept with somewhat different wording, such as individual coaching sessions for parents [41] or ensuring that therapy has both individual and family time [40].

Several studies have also described the role of psychoeducation. This generally focused on teaching about the relationship between autism and EDs [35, 39, 41, 44, 45], with Loizou [39] specifically suggesting the inclusion of topics such as diagnostic overshadowing, delayed diagnosis, and masking (which is the suppression of autistic traits and the adoption of neurotypical behaviours, also known as camouflaging [49]). Two papers described additional psychoeducation around emotions and emotion regulation to support distress tolerance [39, 44].

There were also calls for greater time allowance, although the exact nature differed between studies. Some studies suggested more processing time during therapy [34, 37, 40, 41] while others advocated for a more gradual pace or longer treatment timeline more generally [34, 35, 40, 43].

### Adaptations that may Challenge FT-ED

A small body of literature highlights areas where core assumptions of the FT-ED model may be more difficult to apply when working with Autistic young people and their families. Qualitative studies described the use of a more collaborative approach to treatment [34, 35, 37, 38, 40, 43]. Alford et al. suggested that collaboration be considered part of the general foundation for ED treatment with Autistic young people and families [40]. Some studies suggested that Autistic young people be welcomed to take a more active and collaborative role in FT-ED [38, 43], and one study highlighted the importance of informing the young person about what FT-ED is [37]. Some parents and carers preferred to leave some responsibility or choice to the young person [34, 35, 43], a stance which conflicts with the usual approach to early-stage FT-ED where there is an assumption that giving too much choice to the child at this point is unhelpful and uncontaining given the strength of ED cognitions, the effects of starvation, and the high levels of ambivalence present.

Certain principles of the FT-ED manuals, including agnosticism and externalization, have also been challenged. Both parents [34] and clinicians [38] reported difficulties in taking a strictly agnostic view to the cause of the ED when supporting Autistic young people in FT-ED. Further, some clinicians reported that considering autistic needs and traits (e.g., sensory sensitivities, social preferences) within the initial formulation, and how these interact with the ED, helped to support subsequent treatment adaptations [38, 43]. Additionally, multiple studies found that the use of externalization, another key approach used in FT-ED, did not always resonate with Autistic family members [34, 38, 40, 43].

### Adjunct Interventions

Some studies responded to the perceived limitations of FT-ED with Autistic young people by providing adjunct sessions. Loizou [39] offered a once-weekly group session for parents of young people (age 12–17) who were diagnosed or suspected autistic and participating in FT-ED. The seven sessions occurred during the middle and ending phases of FT-ED and included psychoeducation on autism and AN, emotion coaching/distress tolerance, sensory needs, parental self-care, and tolerating endings. Of the 17 parents (from eight families), the attendance rate was 66.4% across all sessions, with at least one parent from each family attending 85.7% of sessions. With ratings out of ten, sessions were considered relevant (M = 8.61, SD = 1.60), useful (M = 8.58, SD = 1.63), and helpful in improving understand of their child (M = 7.71, SD = 2.18).

Holliday [41] described low-intensity adjunct sessions for young people, including a three session modular intervention to support understanding and advocating for sensory needs, as well as a four session intervention—Finding Connections—which supports unmasking and findings ways to connect to others as your authentic Autistic self. Two studies also provided lived experience ideas for future adjunctive therapies. This included some young people suggesting music or art therapy as an outlet for self-expression [37] and some parents suggesting peer support to connect with other parents [35].

## Reflections and Future Directions

Studies reporting on the use and outcomes of FT-ED with Autistic young people or those with high autistic traits generally reached similar rates of remission as non-autistic young people or those with low autistic traits, but through higher rates of service use, such as longer or more intensive treatment. Parental experiences of FT-ED with an Autistic young person included the need to be an advocate for their child, particularly when autistic traits were pathologized as ED behaviours. Young peoples’ experiences highlighted a lack of voice within FT-ED, although increased collaboration and autism-affirming adaptations seemed to improve these experiences. Clinicians considering FT-ED adaptations for Autistic young people often reported anxiety and concern about veering from the evidence base. Many potential adaptations to FT-ED to make care more autism-affirming were reported, including environmental adjustments (e.g., quiet spaces, dimmed lights), sensory-informed understanding of food and eating preferences (e.g., accounting for historical eating behaviour), communication adaptations (e.g., passports and clear, literal language), psychoeducation on autism and EDs, careful consideration of externalization, and use of separated sessions. However, the literature on adaptations was mainly limited to qualitative and descriptive research.

When considering the experiences of FT-ED reported in the qualitative literature by Autistic young people, their parents/carers, and their clinicians, it is important to note that some of these findings mirror existing research around FT-ED with the general population. For example, previous research has found that some young people partaking in FT-ED for AN felt that their psychological distress was neglected, and that their voice was not heard [50]. Similarly, some FT-ED clinicians reflected that strict fidelity to a manual restrained them from tailoring treatment to the unique young person and family [51]. Therefore, it is unclear which themes raised in the autistic qualitative literature are unique to aspects of being autistic and which themes might be universally applicable—but perhaps more likely to occur—in autistic populations, or why this might be.

When considering the proposed autism-affirming adaptations of FT-ED for AN, it is relevant to consider which represent novel recommendations and which may be reflected in the evolving nature of FT-ED. For example, FT-AN has become more collaborative over time, now including the practice of individual formulation, and considers a broader concept of externalisation [18, 52]. There has also been some exploration of how formulation may fit within the FBT model [20]. Further, the practice of parent/carer only sessions, or holding separate sessions for the young person and the parent/carer, is already recommended as part of the FT-ED model [53, 54], specifically when emotional expression is high [55, 56]. The relative absence of this practice within clinical settings may represent a lack of therapist awareness of this technique rather than a preference for whole family therapy. The development of clinical resources such as treatment guidelines or decision-making tools may support the dissemination and implementation of autism-affirming adaptations—both those that are completely novel and those that link to the evolving FT-ED model.

Future research may wish to more firmly evaluate the impact of FT-ED and potential adaptations on Autistic young people using quantitative approaches. Historically, clinical trials in FT-ED for AN have not screened for autism/autistic traits [24]. There has been a recent call for more randomised control trials in the treatment of children and young people with AN [29], and these future trials need to examine outcomes specifically for Autistic individuals. There is also growing recognition of the role of routine outcome measurement and clinical quality registers in improving ED treatment by systematically collecting data during routine clinical care [57]. Existing frameworks for this data collection recommend the measurement of autism diagnosis or autistic traits [58–60]. While this has started to emerge [29], services currently establishing a program of routine outcome measurement may wish to prioritise autism/autistic traits. Finally, pilot studies exploring the impact of suggested FT-ED adaptations, such as the addition of adjunct supports like creative/art therapy or peer support, may be a feasible place to start the exploration of the initial acceptability and effectiveness of autism-affirming adaptations.

Future research may also wish to explore the experiences of young people with bulimia nervosa (BN). While most of the available research focuses on the outcomes, experiences, and suggested adaptations to FT-ED for those with AN, recent evidence suggests that neurodivergence is common amongst individuals with bulimia nervosa (BN) [61]. FT-ED has been developed for young people with BN [62, 63], yet there are no studies currently available on the experiences of these Autistic young people, their parents/carers, or their clinicians.

## Conclusions

This review explores the outcomes and experiences of Autistic children and young people, parents and carers, and their clinicians in relation to FT-ED for AN. The qualitative research suggests a range of potential autism-affirming adaptations to FT-ED, but it is important to note that, from the wide array of treatment adaptations suggested, one size will not fit all. Spending additional time on formulation and considering the impact of autistic profiles on the maintenance of the ED, as well as harnessing autistic strengths to promote recovery, may best support Autistic young people and their family in the journey through FT-ED.

## Data Availability

No datasets were generated or analysed during the current study.
